# Focal and Steerable Temporal Interference Stimulation of the Human Left Posterior Insula: A Proof-of-Concept Validation Using Intracranial Recordings in a Human Cadaver

**DOI:** 10.3390/bioengineering13091050

**Published:** 2026-09-10

**Authors:** Yaser Fathi, Aïcha Boutachkourt, Vincent Joris, Valeria Onofrj, Laurence Dricot, Catherine Behets, Bernard Hanseeuw, Giulia Liberati

**Affiliations:** 1 Institute of Neuroscience, Université Catholique de Louvain, 1200 Brussels, Belgium; 2 Department of Neurosurgery, Cliniques Universitaires St-Luc, 1200 Woluwe-St-Lambert, Belgium; 3 Department of Radiology, Cliniques Universitaires Saint-Luc, 1200 Bruxelles, Belgium; 4 Pole of Morphology, Institut de Recherche Expérimentale et Clinique (IREC), UCLouvain, 1200 Brussels, Belgium; 5 Neurology Department, Cliniques Universitaires Saint-Luc, 1200 Brussels, Belgium; 6 Psychological Sciences Research Institute, UCLouvain, 1348 Louvain-la-Neuve, Belgium

**Keywords:** temporal interference stimulation (TIS), noninvasive brain stimulation (NIBS), insular cortex, electric field modeling, neuromodulation

## Abstract

The insular cortex, especially its posterior subdivision, is a key hub for interoceptive, somatosensory, and nociceptive processing, making it a compelling target for neuromodulation. However, its depth within the lateral sulcus makes it difficult to reach selectively with conventional noninvasive brain stimulation. Temporal interference stimulation (TIS) can, in theory, focus low-frequency modulation onto deep structures, but experimental validation for the human insula is lacking. We tested whether a simple, simulation-guided TIS approach can focally and steerably modulate electric fields within the human insula. Using the TI Planning Tool, we designed anterior–posterior (AP) optimized, lateral-optimized, and non-optimized control montages and recorded induced fields from six intracranial depth electrodes spanning insular subregions in a fresh-frozen donated human body. TIS was delivered at 990 and 1000 Hz (10 Hz envelope) across five current-ratio conditions. The AP-optimized montage produced preferential envelope modulation in deep posterior insular contacts, an effect robust across contact-depth thresholds and stronger than for absolute field amplitude. Whereas varying the current ratio steered the modulation focus monotonically along the AP axis, control montages showed no such steering. These findings provide proof-of-concept evidence that simulation-guided TIS can focally target and steer stimulation within the human posterior insula.

## 1. Introduction

**Figure 1:**
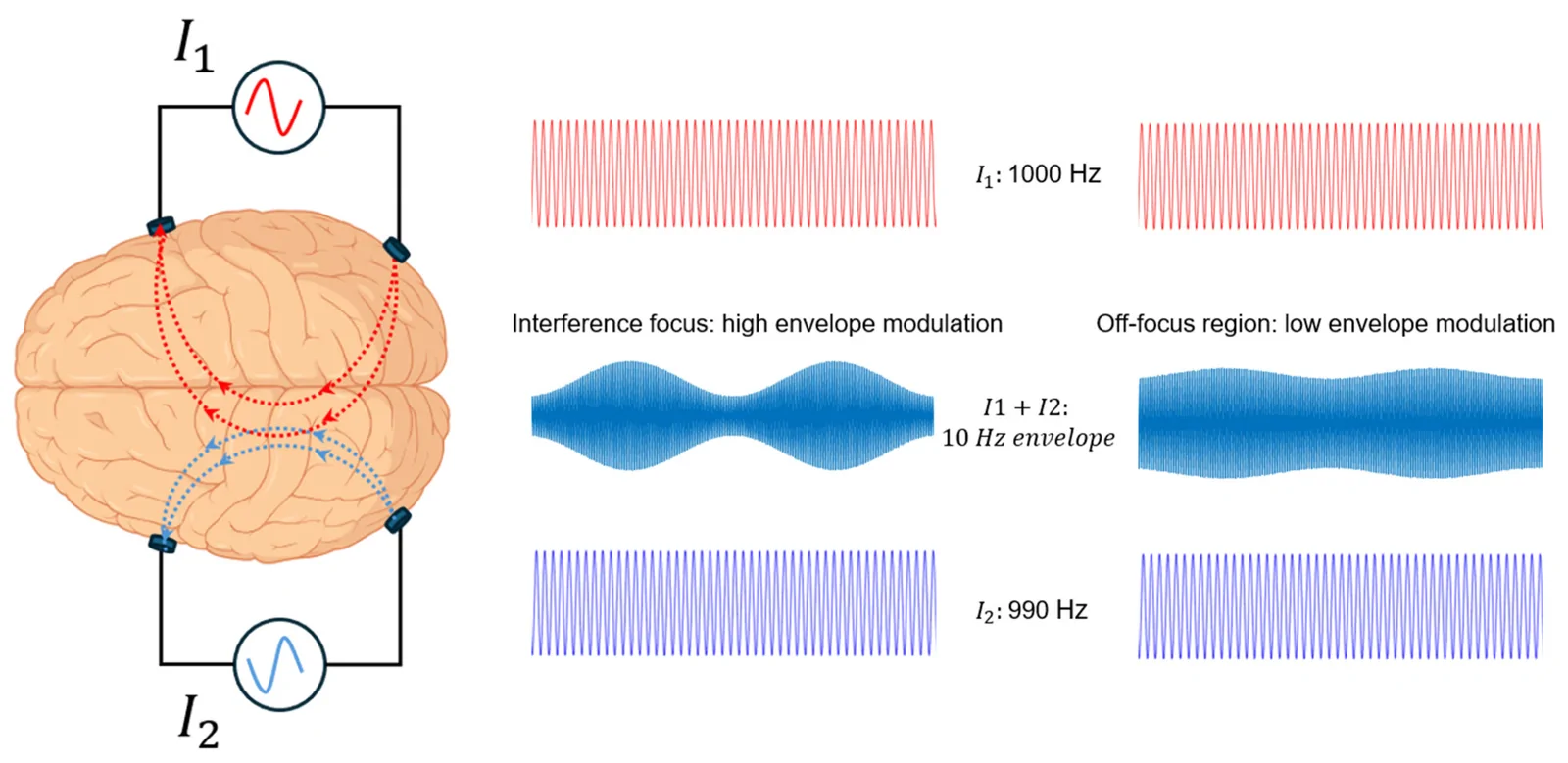
Schematic illustration of the principle of temporal interference stimulation (TIS). Two high-frequency currents, $I_{1}$ at 1000 Hz and $I_{2}$ at 990 Hz, generate a combined signal with a 10 Hz amplitude envelope. Envelope modulation is higher at the interference focus and lower in off-focus regions.Red and violet traces represent the two high-frequency currents I_1_ (1000 Hz) and I_2_ (990 Hz); blue traces represent their superposition, whose amplitude envelope oscillates at 10 Hz. On the brain schematic, red and blue dotted arrows indicate the current paths of the first and second electrode pairs.

Conventional noninvasive brain stimulation techniques—such as transcranial magnetic stimulation (TMS) and transcranial alternating current stimulation (tACS)—are generally limited in their ability to selectively modulate deep or buried cortical regions without simultaneously affecting overlying cortical areas [1]. Temporal interference stimulation (TIS) has emerged as a promising approach to address this limitation [2]. By applying two high-frequency electrical currents with slightly different frequencies, TIS generates a low-frequency amplitude-modulated envelope that can be spatially separated from regions of maximal electric field intensity (Figure 1). Computational studies and a limited number of experimental investigations have suggested that TIS may improve the targeting of deep brain structures while reducing stimulation of the surrounding tissues. A recent systematic review of human TIS studies further highlighted both the promise of this approach for noninvasive deep brain modulation and the need for additional experimental validation of its perisylvian-targeting capabilities [3].

The insular cortex is a deeply buried cortical structure located within the lateral sulcus and is involved in a broad range of functions, including interoception, somatosensory processing, emotion, autonomic regulation, and higher-order cognition [4,5]. This functional diversity is supported by its heterogeneous anatomical and functional organization, with anterior and posterior subdivisions showing distinct patterns of connectivity and functional specialization [6].

Within this organization, the posterior insula is of particular interest as a target for neuromodulation because of its role in representing bodily states and processing interoceptive and somatosensory information, including temperature, touch, visceral sensations, and nociception [7]. Beyond sensory processing, altered structure or functional connectivity of the posterior insula has also been reported in major depressive disorder [8] and addiction [9], supporting its broader relevance to neuropsychiatric function. Functional specialization within the insula suggests that posterior regions are more strongly associated with bodily and sensory processing, whereas anterior regions contribute more prominently to affective and higher-order integration [6].

Furthermore, the insula is widely recognized as a key cortical site for interoceptive and nociceptive processing [10,11,12,13], and intracerebral EEG recordings from our group have demonstrated that nociceptive stimulation preferentially modulates theta and alpha oscillatory activity within the dorsal posterior insula [14]. Evidence from lesion studies further supports its importance, showing that damage to the posterior insula can profoundly alter the subjective experience of pain while preserving basic sensory detection [15]. Together, these findings identify the posterior insula as a compelling neuromodulation target and motivate the development of approaches capable of selectively engaging this deep cortical region.

However, despite its importance, the posterior insula remains challenging to reach selectively with conventional noninvasive stimulation. Recent modeling work has further suggested that the insula represents a feasible target for TIS, owing to its anatomical location and depth, although experimental validation remains limited [16].

The feasibility of deep-brain targeting with TIS has been supported by postmortem human studies combining computational modeling with intracranial measurements [17,18]. Acerbo and colleagues demonstrated preferential targeting of the hippocampus using scalp electrodes in human cadavers, reporting greater envelope modulation amplitudes within the hippocampus than in more superficial cortical regions [17]. Similarly, Violante and colleagues showed in human cadavers that temporal interference fields could be focused and steered within the hippocampus, with intracranial recordings revealing stronger modulation in the hippocampus than in the overlying cortex and demonstrating that the location of maximal modulation could be shifted through adjustments of the applied currents [18]. These findings provided important experimental support for the concept of steerable deep-brain targeting with TIS and established postmortem human recordings as a valuable intermediate step between computational modeling and in vivo application.

Although these cadaver studies established the feasibility of steerable deep-brain targeting with TIS, they focused on the hippocampus, and no equivalent experimental validation exists for the human insula. The insula poses a distinct and arguably greater challenge: it is a folded cortical structure buried deep within the lateral sulcus and surrounded by densely packed opercular and vascular tissue, so that selectively concentrating an interference envelope within a specific insular subregion, rather than within a compact subcortical nucleus, cannot be assumed from previous hippocampal results. In the present proof-of-concept postmortem study, we therefore investigated whether a simple, simulation-guided TIS approach can selectively modulate electric field distributions within the human insular cortex, with a particular focus on the left posterior insula. Specifically, we aimed to evaluate (1) whether optimized TIS montages could produce preferential envelope modulation within posterior insular regions and (2) whether the location of maximal modulation could be steered along the anterior–posterior insular axis by varying stimulation current ratios. By combining computational planning with intracranial recordings from a donated human body, this study provides an initial biophysical assessment of the feasibility of targeting the human insula with TIS and of resolving distinct locations along its anterior–posterior axis.

## 2. Materials and Methods

### 2.1. Electrode Configuration Setup for Stimulation

Electric field simulations for TIS were performed using the TI Planning Tool (TIP.lite, IT’IS Foundation, https://tip-lite.science (accessed on 4 September 2026)), a software platform that estimates the spatial distribution of amplitude-modulated electric fields in predefined anatomical head models. The software uses a highly detailed anatomical head called MIDA, which is derived from multimodal MRI data and incorporates detailed tissue segmentation and anisotropic conductivity maps [19]. We used the classic TI mode of the software, in which two high-frequency sinusoidal currents with slightly different frequencies are applied. The TIP considers three key objectives: (1) target strength, defined as the median modulation amplitude within the target region; (2) selectivity, the ratio of mean exposure in the target relative to the rest of the brain; and (3) collateral stimulation, the fraction of off-target brain receiving stimulation above the target median. The relative importance of target strength, selectivity, and collateral stimulation was specified using the software’s weighting parameters, which can independently vary between 0 and 1. The corresponding weights were set to 0.5, 1.0, and 0.5, respectively. Selectivity was therefore weighted twice as heavily as target strength, prioritizing the focality of the modulation envelope over the amplitude delivered to the target.

In the simulations, we used circular scalp electrodes with a surface area of 3.14 cm^2^. The left insula was defined as the target region. For each electrode pair, we selected multiple candidate positions using the tool’s interactive interface. We then refined these candidates iteratively over several runs, comparing the output metrics—target strength, selectivity, and collateral stimulation—to identify the final electrode configuration.

**Figure 2:**
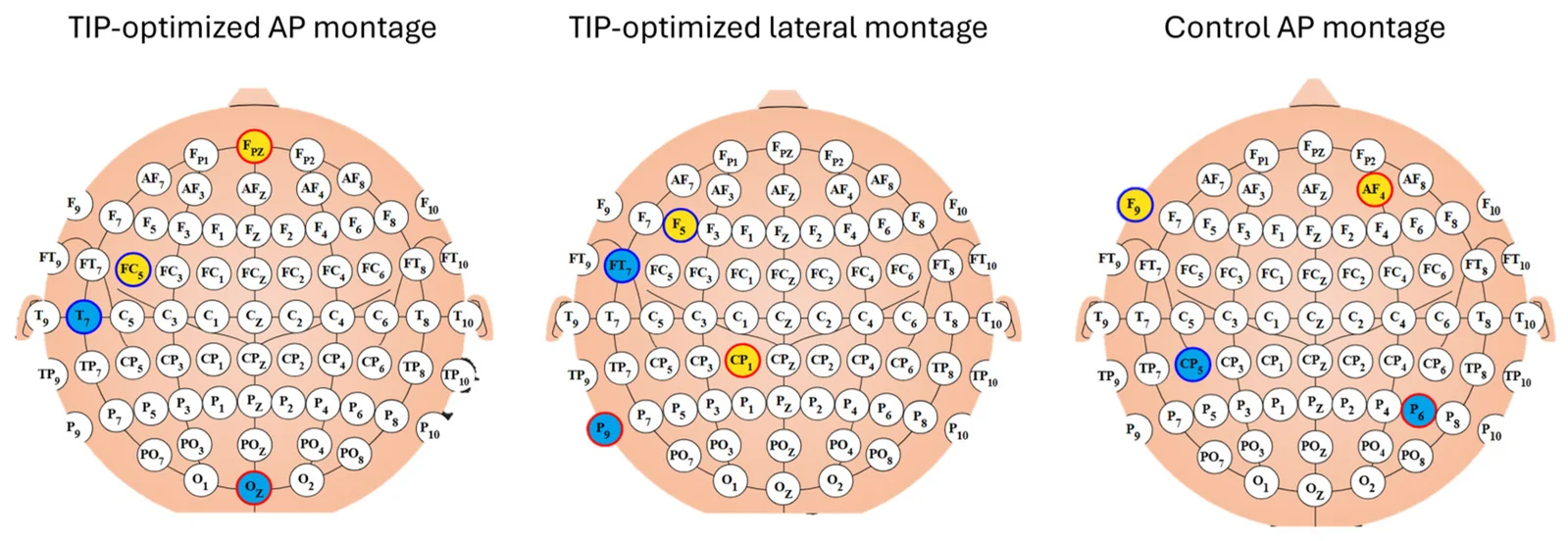
Electrode montages used for temporal interference stimulation (TIS). The left panel shows the TIP-optimized anterior–posterior (AP) montage, in which the two electrode pairs were positioned along the anterior–posterior axis based on simulation-driven optimization. The middle panel shows the TIP-optimized lateral montage, where the electrode pairs were arranged predominantly along the lateral axis while maintaining optimization by the TIP framework. The right panel shows the control AP montage, consisting of an anterior–posterior electrode arrangement that was not selected based on optimization criteria and served as a control configuration.

Three electrode configurations were evaluated (Figure 2). Two were extracted using a software optimization tool: one aimed to produce I1/I2 interference patterns, sweeping the target region along the anterior–posterior axis (TIP-optimized AP montage), and the other along the medio–lateral axis (TIP-optimized lateral montage). A third non-optimized AP montage was included as a control configuration to benchmark the software-optimized montages: its electrodes were positioned along the same anterior–posterior axis as the optimized AP montage but at locations that were not selected by the optimization procedure, so that any difference in focality or anterior–posterior steering could be attributed to the optimization itself rather than to the general electrode arrangement. This design allowed us to assess whether TIS can be used to focally target the insula and whether the electric field can be steerably modulated. As an additional quantitative comparison with the computational predictions, experimental analogues of the TIP metrics were calculated using anatomically classified target and off-target recording locations (Supplementary Methods and Table S1).

### 2.2. Postmortem Electrical Stimulation and Recording

A fresh-frozen head from a female body donor, aged 91 years at death, was provided through the body donation program of the Faculty of Medicine of UCLouvain (Brussels, Belgium). All procedures were performed in accordance with institutional and ethical guidelines governing the use of donated human bodies for research (ethical approval: UCL-2020-355).

To measure the electric field in deeper targets, induced by noninvasive electrical stimulation, after thawing, intracranial depth electrodes were implanted by an experienced neurosurgeon (V.J.) following clinical implantation procedures commonly used in epilepsy patients. Six Microdeep electrodes (Dixi Medical, Besançon, France) were used, each with a diameter of 0.8 mm and containing a variable number of recording contacts (8, 10, or 15), with each contact measuring 2 mm in length and spaced 1.5 mm apart. A craniotomy was performed over the perisylvian regions to allow implantation of the depth electrodes. Direct exposure through sylvian fissure dissection was not performed to avoid tissue damage. Electrodes were thus implanted into different regions of the insular cortex by a transopercular trajectory. The placement was as follows: a 10-contact electrode was positioned in the anterior insula; a 10-contact electrode was placed in the posterior insula; a 15-contact electrode was implanted in the middle insula; an 8-contact electrode was placed in the middle-anterior insula; an 8-contact electrode was positioned in the middle-posterior insula; another 8-contact electrode was inserted into the dorsal insula. The bone flap was then repositioned and the skin sutured to restore the macroscopic cranial anatomy as closely as possible to the intact condition before stimulation and recording.

The ground electrode was placed close to the mastoid using an ECG electrode (Kendall™ H124SG, Cardinal Health, Dublin, OH, USA, 30 × 24 mm). A 64-channel EEG recording system with 4 kHz sampling rate and active shielding (ANT Neuro, Enschede, The Netherlands) was used to record electric potential signals induced by stimulation.

To deliver TIS, we used two electrical current sources (DS5, Digitimer Ltd., Welwyn Garden City, Hertfordshire, UK), each driven by voltage waveforms generated by separate NI USB DAQ devices, which were controlled independently by two laptops. TIS was generated using sinusoidal currents at 990 and 1000 Hz, producing a 10 Hz envelope modulation frequency. Carrier frequencies in the 1–2 kHz range are the standard choice in human TIS studies [3], and 1 kHz carriers with a low-frequency envelope have been used both in TIS modeling work [20] and in phantom measurements of the interference field recorded with depth electrodes [21]. Carriers in this range remain well above the range of endogenous neural frequencies (>600 Hz), so that they are not expected to directly entrain neural activity, while still being transmitted efficiently through skull and brain tissue [20,22]. The difference frequency was set to 10 Hz, within the alpha band. A baseline amplitude of 1.0 mA per source was selected in line with previous human-cadaver measurements [18]. Five reciprocal ratios around 1:1 were tested to assess graded bidirectional steering, following a previous study that shifted the location of maximal modulation by varying the relative amplitudes of the two sources [18]: 1:2 (0.8 and 1.6 mA), 2:3 (0.8 and 1.2 mA), 1:1 (1.0 and 1.0 mA), 3:2 (1.2 and 0.8 mA), and 2:1 (1.6 and 0.8 mA). All current amplitudes reported here are zero-to-peak values. These current ratios were selected to evaluate steering of the interference pattern while maintaining stimulation intensities within the operating range of the stimulation system. The 2:3 and 3:2 conditions maintained a total current of 2.0 mA. Maintaining this total for 1:2 and 2:1 would have required reducing one source to 0.67 mA; because outputs below approximately 0.75 mA showed waveform distortion in our stimulation chain, these conditions were implemented as 0.8/1.6 mA, resulting in a total current of 2.4 mA. Each current source was delivered through the scalp using a pair of electrodes (Kendall™ H124SG, Cardinal Health, 30 × 24 mm). Electrodes were positioned according to configurations determined in the previous step, as described earlier. For each montage and current-ratio condition, intracranial recordings were acquired during 25 s stimulation periods. Conventional tACS recordings were also acquired using each of the three montage configurations. Sinusoidal currents at 10 Hz and 1 mA zero-to-peak were delivered through the same electrode pairs used for TIS. Each tACS condition was applied for 25 s.

### 2.3. MRI Scanning for Validation of Implanted Electrode Locations

Following electrode implantation, post-implantation magnetic resonance imaging (MRI) was performed on a 3 T SIGNA™ Premier MRI scanner (GE Healthcare, Milwaukee, WI, USA) equipped with a 48-channel head coil to verify the anatomical locations of the implanted depth electrodes. A high-resolution three-dimensional T1-weighted magnetization-prepared rapid gradient-echo (MPRAGE) image was acquired using the following parameters: repetition time (TR) = 2188.16 ms, echo time (TE) = 2.96 ms, inversion time (TI) = 900 ms, 156 sagittal slices, isotropic voxel size = 1 × 1 × 1 mm^3^, and field of view (FOV) = 256 × 256 mm^2^. DICOM images were converted to the NIfTI format using MRIcroGL for subsequent processing and visualization. The MRI data were then imported into BrainVoyager (Brain Innovation, Maastricht, the Netherlands) and transformed into the Talairach space to facilitate standardized anatomical localization. The position of each electrode contact was identified on the post-implantation MRI using anatomical landmarks, and Talairach coordinates were extracted. Anatomical labels were assigned according to the Talairach atlas and further verified by visual inspection of the surrounding anatomical structures to ensure accurate localization of the recording contacts (Table S2).

### 2.4. Data Analysis

Signal processing followed the general approach described by Violante et al. [18]. Intracranial recordings were segmented into 25 s stimulation periods corresponding to each stimulation condition. To remove slow drifts, each channel was detrended using a third-order polynomial. Signals were then transformed into an adjacent bipolar montage by subtracting neighboring contacts along each electrode shaft, ordered from the deepest to the most superficial contact. This finite-difference approach was used to reduce common-mode signals and reference-dependent effects and to obtain a local voltage-gradient measure along each electrode trajectory, which approximates the electric field component along the shaft.

Bipolar signals were notch-filtered at 50 Hz and its harmonics (100 and 150 Hz). For TIS recordings, signals were band-pass filtered around the carrier-frequency range (500–1500 Hz), whereas conventional tACS recordings were band-pass filtered between 1 and 40 Hz. The amplitude envelope was extracted using the endpoint-corrected Hilbert transform and subsequently low-pass filtered to isolate the low-frequency modulation component.

Signals were divided into consecutive 1 s epochs. For each bipolar channel, the absolute signal amplitude was quantified as the median of the epoch-wise maximum amplitudes. Envelope modulation strength was quantified for TIS recordings, as the mean half peak-to-peak amplitude of the extracted envelope across epochs:

$$A_{\mathrm{env}}=\frac{1}{N}\overset{N}{\underset{i=1}{\sum }}\frac{\max\left(E_{i}\right)-\min\left(E_{i}\right)}{2}$$

where $E_{i}$ denotes the extracted envelope during epoch i, and N is the total number of epochs.

To facilitate comparison across electrode shafts with different numbers of contacts, both absolute amplitude and envelope modulation amplitude were normalized independently within each shaft to the maximum value observed along that shaft. The resulting profiles ranged from 0 to 1 and represent relative spatial distributions of the local voltage-gradient measure rather than absolute electric field strength. For visualization, normalized profiles from each electrode trajectory were interpolated to a common normalized contact-position axis and displayed as two-dimensional maps showing the spatial distribution of absolute amplitude and envelope modulation amplitude across the six planned insular electrode trajectories (Ant_10, Ant_8, Spr_8, Mdl_15, Post_8, and Post_10). For the glass-brain visualization, normalized envelope modulation and absolute amplitudes were mapped to the Talairach coordinates of individual contacts and displayed on a glass-brain template using BrainNet Viewer in MATLAB R2023a (MathWorks, Natick, MA, USA) [23].

To assess depth-dependent spatial selectivity, each electrode trajectory was divided into deep and superficial portions using a predefined deep-contact ratio (R = 0.5). The deepest 50% of contacts were classified as deep contacts, and the remaining contacts as superficial contacts. Electrode trajectories were grouped according to their predefined implantation configuration and relative anteroposterior position. Ant_8 and Ant_10 constituted the anterior trajectory group, whereas Spr_8, Mdl_15, Post_8, and Post_10 constituted the posterior-targeting trajectory group. Group comparisons were performed separately for normalized envelope modulation amplitude and normalized absolute amplitude using two-sided two-sample *t*-tests. Effect sizes were quantified using Hedges’ *g*, with positive values indicating greater amplitudes in posterior trajectories. The 12 *p*-values arising from the three montages, two depth groups, and two amplitude measures were adjusted together using the Bonferroni correction. To address the unequal numbers of anterior and posterior trajectories, an additional analysis was restricted to the matched Ant_10 and Post_10 trajectories. The same analysis was performed at the prespecified deep-contact ratio of R = 0.5. Sample sizes, descriptive statistics, Hedges’ g, raw *p*-values, and Bonferroni-adjusted *p*-values for the primary and matched analyses are reported in Tables S3 and S4, respectively. As a sensitivity analysis, the deep-contact ratio was varied from 0.1 to 0.9. At each ratio, one-sided two-sample *t*-tests evaluated the directional hypothesis that amplitudes were greater in posterior than anterior deep contacts. The resulting *p*-values were adjusted together using the Bonferroni correction.

To evaluate steering of the stimulation focus along the anterior–posterior axis, normalized envelope modulation amplitudes were extracted from the anterior (Ant_10) and posterior (Post_10) electrode trajectories. For each stimulation condition, an anterior-to-posterior (Ant/Post) modulation ratio was calculated as the ratio of the mean normalized envelope modulation amplitudes measured across contacts within the anterior and posterior trajectories. The relationship between stimulation current ratio and the Ant/Post modulation ratio was assessed using Spearman rank correlation. To assess steering independently of total current, additional comparisons were performed within the constant-total-current pairs, 1:2 versus 2:1 at 2.4 mA and 2:3 versus 3:2 at 2.0 mA (Supplementary Table S5).

## 3. Results

**Figure 3:**
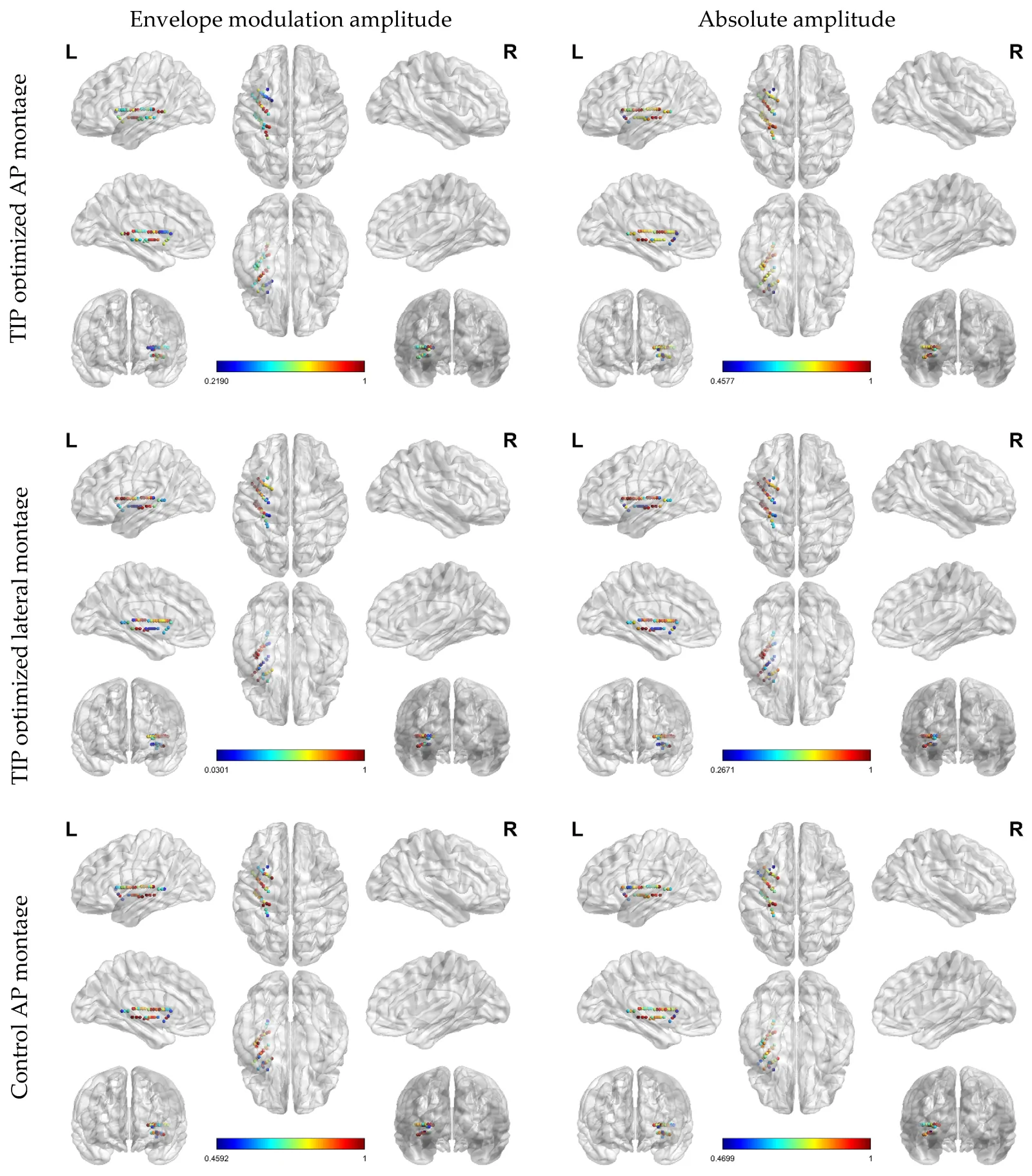
Spatial distribution of recorded amplitudes across intracranial EEG contacts. Glass-brain visualizations generated using BrainNet Viewer in MATLAB show the envelope modulation amplitude (**left column**) and absolute amplitude (**right column**) for the TIP-optimized anteroposterior montage (**top**), TIP-optimized lateral montage (**middle**), and control anteroposterior montage (**bottom**). Contact values are displayed from multiple anatomical viewpoints, with L and R indicating the left and right hemispheres. Colors represent amplitudes normalized within each panel, with 1 corresponding to the maximum value. Only contacts with available anatomical coordinates are shown (40 of 59 contacts). TIP, temporal interference planning tool; AP, anteroposterior; L, left; R, right.

The spatial distributions of normalized envelope modulation amplitude and normalized absolute amplitude across the six insular electrode trajectories are shown in Figure 3. For the TIP-optimized AP montage, envelope modulation amplitude was preferentially distributed within the deeper portions of the posterior-insular trajectories. In contrast, the TIP-optimized lateral montage and the control AP montage exhibited less pronounced depth-dependent patterns. Differences between the spatial distributions of envelope modulation amplitude and normalized absolute amplitude were most evident for the TIP-optimized AP montage, whereas the two measures showed more similar spatial patterns for the TIP-optimized lateral and control AP montages. These observations suggest greater spatial selectivity of envelope modulation for the TIP-optimized AP montage compared with the other stimulation configurations. Conventional tACS using the same three montage configurations showed broadly similar spatial amplitude patterns to the corresponding TIS absolute-amplitude distributions (Figure S1).

**Figure 4:**
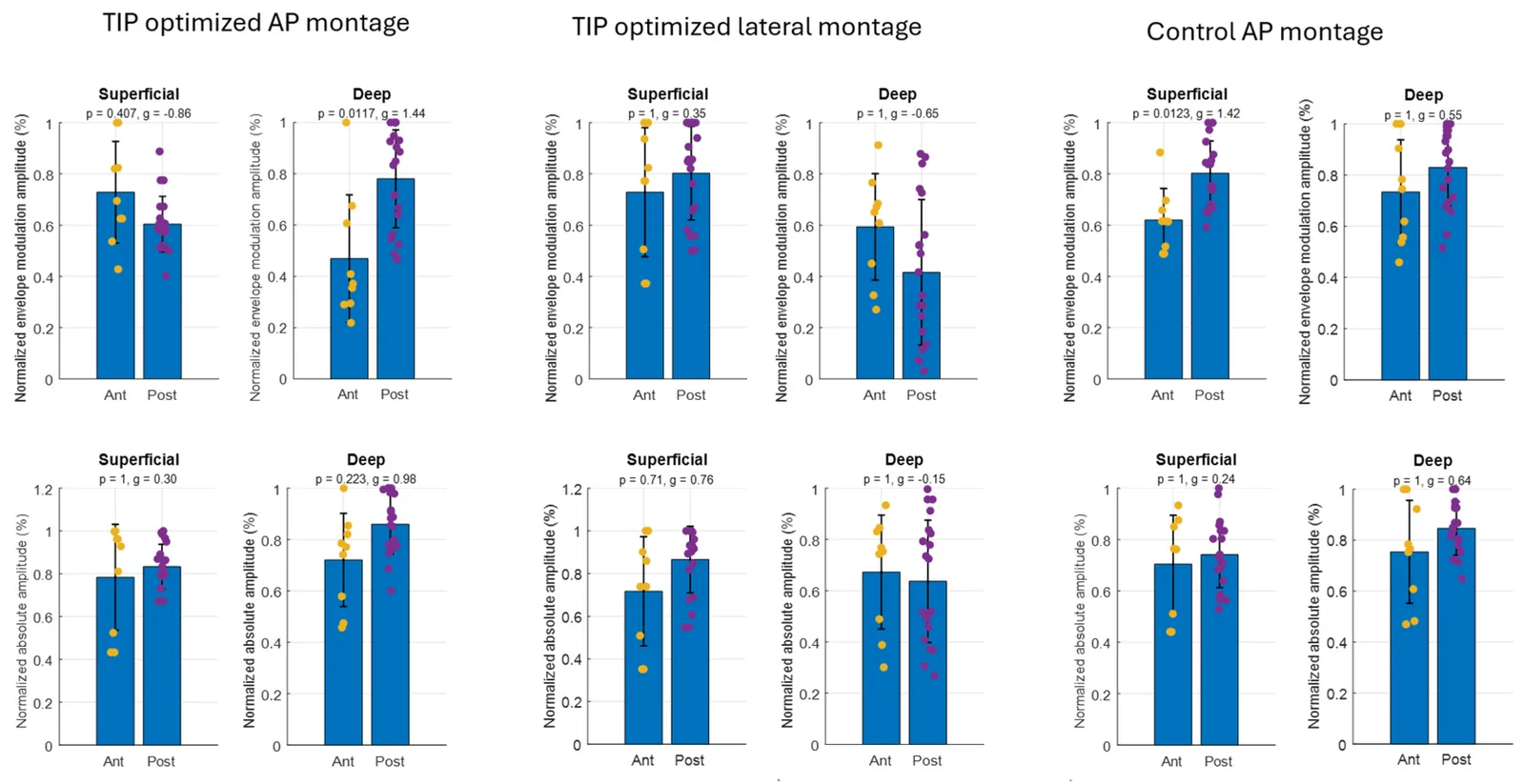
Comparison of normalized envelope modulation amplitude (**top row**) and normalized absolute amplitude (**bottom row**) between anterior (Ant) and posterior (Post) electrode trajectories for the three stimulation montages. For each montage, analyses were performed separately for superficial contacts (**left panel**) and deep contacts (**right panel**), defined as the superficial and deepest 50% of contacts along each electrode trajectory, respectively. Bars represent mean ± SD, and individual points represent contacts. *p*-values were Bonferroni-adjusted across the 12 comparisons. Effect sizes are reported as Hedges’ g, with positive values indicating Post > Ant. A total of 18 anterior and 41 posterior contacts were included. Ant, anterior; Post, posterior; TIP, temporal interference planning tool; AP, anteroposterior; SD, standard deviation.

To quantify these observations, comparisons were performed between anterior (Ant_8 and Ant_10) and posterior (Spr_8, Mdl_15, Post_8, and Post_10) electrode trajectories. At the prespecified deep-contact ratio of R = 0.5, comparisons were performed separately for superficial and deep contacts (Figure 4). For the TIP-optimized AP montage, posterior trajectories showed greater normalized envelope modulation amplitude than anterior trajectories in deep contacts (p=0.0117, g=1.44). The superficial comparison showed the opposite direction but was not significant (p=0.407, g=−0.86). The TIP-optimized lateral montage showed no significant differences for superficial (p=1.000, g=0.35) or deep contacts (p=1.000, g=−0.65). For the control AP montage, posterior trajectories showed greater envelope modulation amplitude in superficial contacts (p=0.0123, g=1.42) but not in deep contacts (p=1.000, g=0.55). All reported *p*-values were Bonferroni-adjusted. Compared with envelope modulation amplitude, normalized absolute amplitude showed weaker differences between posterior and anterior trajectories. The largest effect was observed for deep contacts in the TIP-optimized AP montage (p=0.223, g=0.98). Complete results for the primary analysis, including raw and Bonferroni-adjusted *p*-values, are provided in Table S3. In the matched Ant_10–Post_10 analysis, deep envelope modulation was greater in Post_10 than Ant_10 for the TIP-optimized AP montage (raw *p* = 0.0028, Bonferroni-adjusted *p* = 0.0333, g = 2.43), whereas no other matched comparison remained significant after correction (Figure S2 and Table S4).

**Figure 5:**
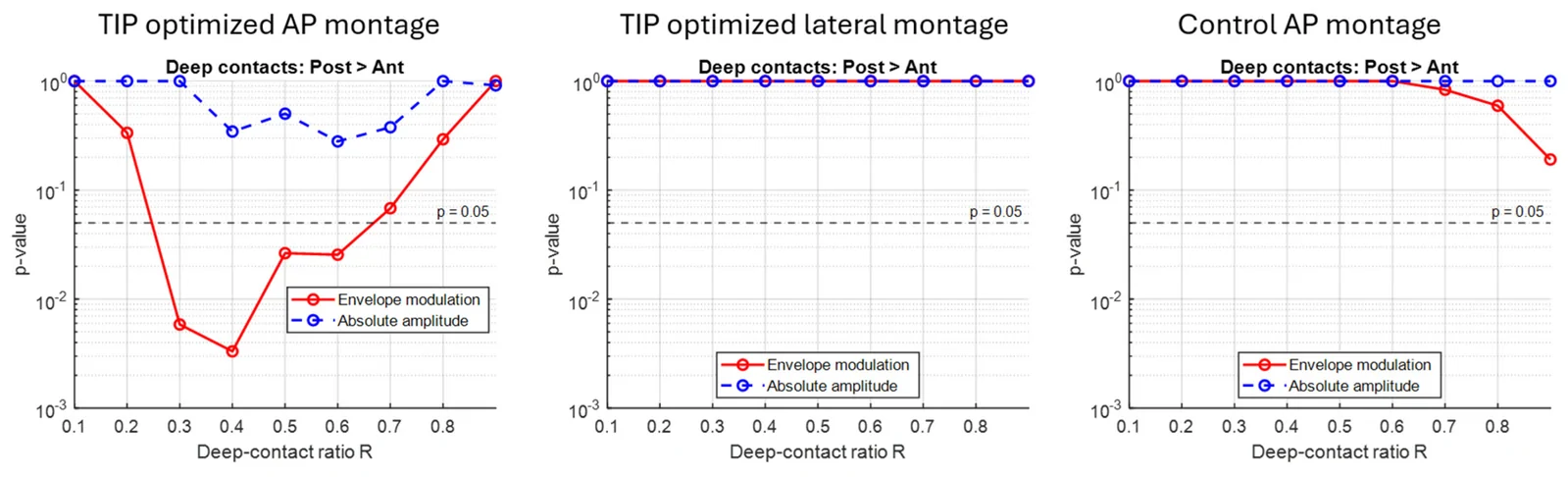
Sensitivity analysis of the deep-contact ratio (R). Bonferroni-adjusted one-sided *p*-values testing the directional hypothesis Post > Ant are plotted against the deep-contact ratio R on a logarithmic scale. Red and blue curves represent envelope modulation and absolute amplitude, respectively. The dashed line indicates p=0.05. A total of 18 anterior and 41 posterior contacts were included, with the proportion classified as deep varying from R = 0.1 to 0.9. Ant, anterior; Post, posterior; TIP, temporal interference planning tool; AP, anteroposterior.

To assess the robustness of the anterior–posterior comparison, the proportion of contacts classified as deep contacts was systematically varied from 10% to 90% (Figure 5). For the TIP-optimized AP montage, posterior trajectories showed significantly greater envelope modulation amplitude than anterior trajectories across deep-contact ratios from R=0.3 to R=0.6. No significant difference was observed for normalized absolute amplitude at any ratio. Neither the TIP-optimized lateral montage nor the control AP montage showed significant posterior–anterior differences for either measure at any tested ratio.

**Figure 6:**
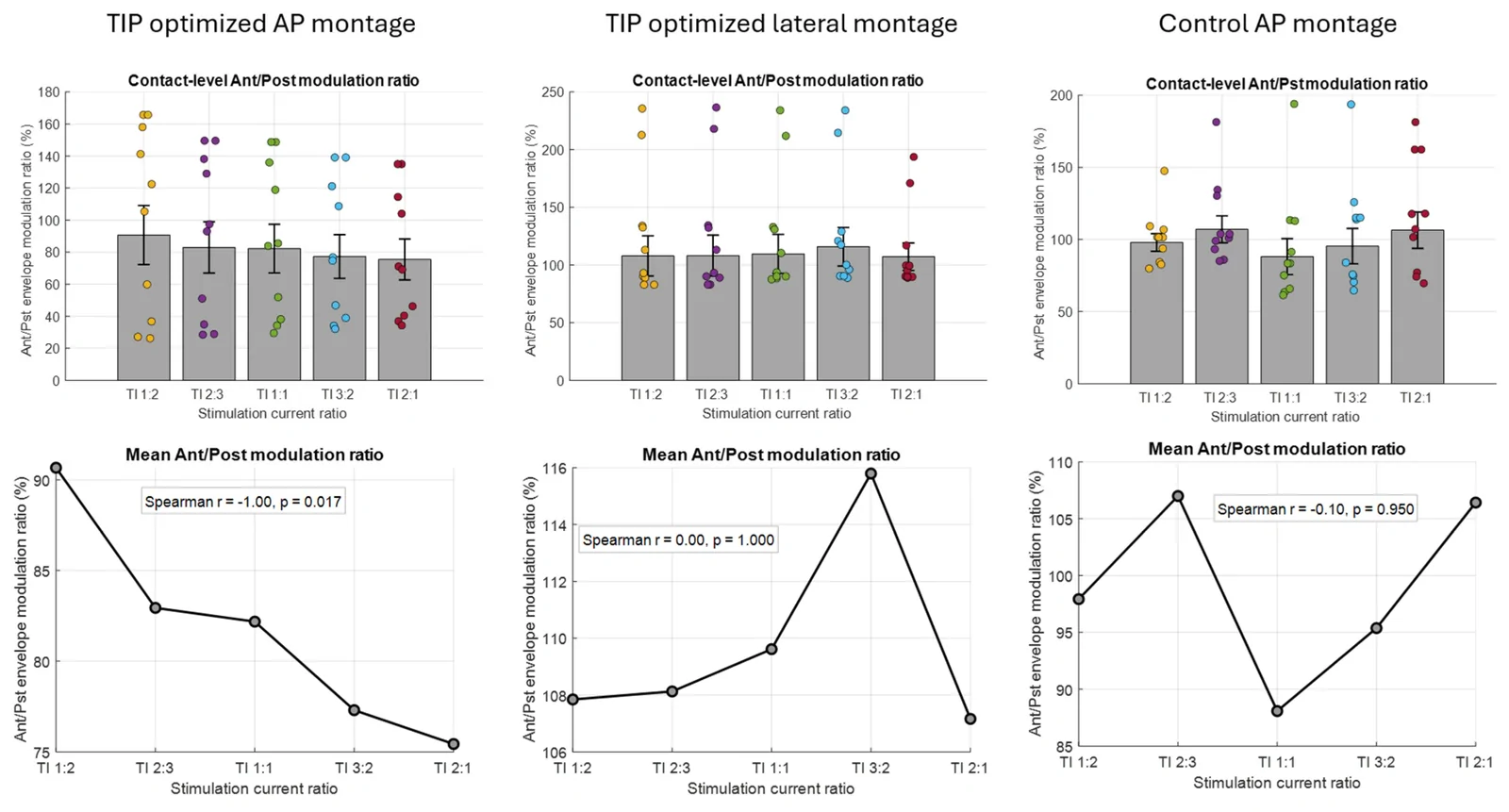
Evaluation of anterior–posterior steerability across stimulation montages. Top panels show contact-level anterior-to-posterior (Ant/Post) envelope modulation ratios for each stimulation current ratio, with bars representing mean ± SEM across contacts. Bottom panels show the corresponding mean Ant/Post modulation ratios as a function of stimulation current ratio. The TIP-optimized anterior–posterior montage exhibited a significant monotonic change in the Ant/Post ratio, consistent with steering of the modulation focus along the anterior–posterior insular axis. No significant steerability was observed for the TIP-optimized lateral montage or the control anterior–posterior montage. Ten anterior and ten posterior contacts were included for each of the five current-ratio conditions. Ant, anterior; Post, posterior; TIP, temporal interference planning tool; AP, anteroposterior; SEM, standard error of the mean.

The ability to steer the location of maximal envelope modulation along the anterior–posterior insular axis was evaluated by computing the anterior-to-posterior (Ant/Post) modulation ratio across stimulation current ratios (Figure 6). To maximize sensitivity to modulation shifts along this axis, the analysis was performed using the most anterior (Ant_10) and most posterior (Post_10) implanted electrode trajectories. For the TIP-optimized anterior–posterior montage, the Ant/Post ratio decreased monotonically with increasing current ratio (Spearman r = −1.00, *p* = 0.017), indicating systematic steering of the modulation focus along the targeted axis. In contrast, no significant relationship was observed for the TIP-optimized lateral montage (Spearman r = 0.00, *p* = 1.00) or the control anterior–posterior montage (Spearman r = −0.10, *p* = 0.95). For the optimized AP montage, constant-total-current comparisons showed that the Ant/Post ratio decreased from 90.66% to 75.43% between 1:2 and 2:1 at 2.4 mA, and from 82.93% to 77.29% between 2:3 and 3:2 at 2.0 mA (Supplementary Table S5). These findings demonstrate that only the TIP-optimized anterior–posterior montage produced predictable modulation shifts along the intended anterior–posterior insular trajectory.

## 4. Discussion

Overall, the optimized AP montage produced the strongest preferential increase in envelope modulation amplitude within deep posterior trajectories, whereas differences in absolute amplitude were smaller and less consistent across montages. This suggests that the optimized AP configuration preferentially enhanced temporal interference modulation in deeper posterior insular regions rather than simply increasing overall field exposure. Importantly, the sensitivity analysis demonstrated that this effect remained significant across a broad range of deep-contact ratios, indicating that the observed anterior–posterior separation was not driven by a specific choice of contact-depth threshold. Together, these findings support the robustness of the optimized AP montage in preferentially modulating posterior insular regions.

Beyond demonstrating preferential modulation of posterior insular regions, the optimized AP montage also enabled systematic steering of the modulation focus along the anterior–posterior insular axis. Varying the relative amplitudes of the interfering currents produced a monotonic shift in the anterior-to-posterior modulation ratio, whereas no such relationship was observed for the optimized lateral or control AP montages. These findings suggest that, in addition to improving spatial selectivity, the optimized AP configuration provides predictable control over the location of maximal temporal interference modulation within the insula. Such steerability may be particularly relevant for future applications targeting distinct insular subregions, which have been associated with different sensory, affective, and cognitive functions.

Future studies should determine whether these targeting properties translate into selective neuromodulatory effects in vivo and whether recently developed temporal interference approaches, such as multipair and phase-controlled stimulation strategies [24], can further enhance the selectivity and steering capabilities demonstrated here.

The present measurements characterize the spatial distribution of TIS-induced envelope modulation rather than the dose delivered, and we therefore do not compare them against neuromodulatory thresholds. For context, the simulated envelope amplitudes at our insular target (0.21–0.29 V/m at 1 mA per source) are comparable to those reported for the hippocampus under equivalent modeling conditions in the study on which our protocol was based, where in vivo application at 2 mA per channel produced physiological and behavioral effects [18].

Any TIS montage involves a trade-off: a montage that concentrates the field tightly on the target generally delivers less field to it, and one that maximizes intensity is less focal. Our optimization resolved that trade-off in favor of focality, weighting selectivity twice as heavily as target strength. In vivo work will need to make this choice deliberately, since the appropriate balance depends on whether the priority is anatomical precision or reaching a physiologically effective intensity. Optimization frameworks are now available that return the full range of possible compromises rather than a single montage based on a fixed weighting [25]. Establishing the intensity required for insular engagement will necessarily require in vivo measurement.

Some limitations should be considered when interpreting these findings. First, the results should be regarded as a proof-of-concept validation, as measurements were obtained from a single fresh-frozen head of a single human body donor. This constraint is shared by previous experimental validations of temporal interference field distributions in human cadavers, which likewise relied on single-specimen measurements [17,18]. Postmortem changes in tissue electrical properties substantially alter the overall magnitude of the induced electric fields, whereas their relative spatial distribution is considerably less affected [26]: the dispersion of the field, as opposed to its global strength, was the measure shown to be preserved (see Supplementary Material in [26]). Our analyses depend only on the latter: depth selectivity and anterior–posterior steering are within-specimen contrasts of shaft-normalized profiles acquired under identical tissue conditions rather than estimates of delivered dose. The magnitude of postmortem effects on intracranial current is nonetheless preparation-dependent and remains poorly constrained. Opitz and colleagues observed an increase in electric-field magnitude postmortem [26], whereas Gamba and Delpy reported a 62% reduction in current reaching the brain after death, associated with increased brain impedance [27]. Freeze–thawing was shown to further alter tissue dielectric properties, although the available measurements were obtained at frequencies well above those used here, and tissue conductivity is itself frequency-dependent [28,29]. The present experiment was not designed to isolate or quantify the effects of postmortem interval, freeze-thawing, or carrier frequency. These factors were common to all experimental comparisons, which reduces their influence on the relative findings. Because the optimized AP, optimized lateral, and control AP montages were measured within the same specimen and through the same recording contacts, donor anatomy cannot account for the differences between montages. It does, however, constrain how far the specific montage geometry and the specific magnitude of steering can be generalized. Modeling work in TIS has shown that the focality achievable at a fixed deep target varies substantially across individuals [20]. For the insula specifically, however, group-level and individually optimized montages did not differ significantly in the proportion of off-target tissue exceeding the stimulation threshold across 60 head models [16]. The opposite conclusion has been reached for deeper targets: Brahma and colleagues report focality losses when a common montage replaces individual optimization, together with a high sensitivity of the achieved focality to montage perturbation [21]. We take this to indicate that the value of individualization is target-dependent rather than uniform, the insula being shallower and more laterally accessible than the subcortical structures examined in that work. We therefore restrict this claim to insular targets.

Two aspects of this specimen dependence deserve discussion. First, what generalizes from these measurements is the feasibility of the effect rather than its parameters. The specific montage coordinates, the magnitude of the anterior–posterior shift, and the axis along which optimization succeeded are properties of this donor’s anatomy and would have to be re-derived for another individual. What the findings indeed support is that reciprocal current ratios can produce a monotonic, bidirectional displacement of the modulation locus within a deep operculo-insular target and that this occurred only for the optimized AP montage and not for the lateral or control configurations. The second aspect is that a 91-year-old donor is not a neutral test case, since age-related skull thinning and increased cerebrospinal fluid volume alter how current reaches the brain. Modeling across 587 healthy older adults aged 51–95 years has shown that the field reaching the brain is inversely related to the degree of atrophy [30]. For field magnitude, this works in a conservative direction, since the amplitudes measured here should be lower than those the same montage would produce in a younger brain at the same injected current.

A second limitation is that the electric-field estimates used to guide montage optimization were derived from a generic head model rather than the donor’s own anatomy. Montage optimization necessarily preceded implantation, and no imaging of the donor was available at that stage: the head was fresh-frozen, and the post-implantation MRI was acquired only after craniotomy and electrode placement. Crucially, both the optimized and the control AP montage were delivered to the same head and were therefore subject to the same mismatch, which cannot explain why one outperformed the other. The advantage we measured should therefore be read as a lower bound on what optimization can achieve rather than as evidence that this montage was optimal for this head. We recommend subject-specific head models and optimization for in vivo application.

A third limitation is that the insula was sampled by only six intracranial electrodes, providing a spatially sparse readout that may not fully capture the field distribution across the structure. The limited number of trajectories and contacts, particularly after subdivision by region and depth, may also reduce statistical power and limit the detection of smaller effects, and nonsignificant findings should be interpreted cautiously. Finally, because the measurements were obtained in a cadaver, they characterize the physical distribution of the induced fields but cannot demonstrate any biological or physiological neuromodulatory effect. Confirming functional engagement of the insula will require in vivo studies.

It is relevant to note that only the left insula was targeted and measured. This limitation operates at two levels. At the biophysical level, the delivered field depends on head geometry and tissue conductivity, which are broadly symmetric, so comparable depth selectivity and steerability should be achievable for the right insula following re-optimization; indirect support comes from the group-level insular montage derived across 60 head models comprising 30 individuals and their mirrored counterparts, which performed comparably across them [16]. At the functional level, insular contributions to nociception and interoception have been reported to be asymmetric. In a large intracranial stimulation series, painful sensations were evoked from the upper posterior insula predominantly in the right hemisphere, whereas non-painful somaesthetic sensations were elicited bilaterally [31]. For interoception, the left and right insula have been associated with parasympathetic and sympathetic representation, respectively [32]. While the evidence is not uniform, it is sufficient to caution against treating the two hemispheres as interchangeable functional targets. The present study contains no physiological or perceptual measurement and cannot address this, and hemisphere selection in future in vivo work should follow from the functional target rather than from field considerations alone.

Electrode localization should also be interpreted cautiously because the postmortem brain showed substantial depth displacement relative to the standard Talairach template. Nevertheless, posterior targeting was supported by the planned surgical trajectories and post-implantation MRI, while the focality and steering analyses relied primarily on relative contact positions and within-specimen comparisons rather than exact atlas coordinates.

Overall, this postmortem validation study provides experimental support for the feasibility of selectively targeting posterior insular regions using an optimized temporal interference montage. By demonstrating preferential envelope modulation and predictable steering of the modulation focus through intracranial recordings from a donated human body, this study helps bridge the gap between computational predictions and the experimental validation of temporal interference field distributions.

## Data Availability

The data presented in this study are available on request from the corresponding author.

## Supplementary Materials

The following supporting information can be downloaded at: https://www.mdpi.com/article/10.3390/bioengineering13091050/s1, Supplementary Methods: Quantitative comparison of TIP simulation and intracranial recordings; Figure S1: Comparison of normalized absolute-amplitude distributions during TIS and conventional tACS across the three stimulation montages; Figure S2: Comparison of normalized envelope modulation amplitude and normalized absolute amplitude between the matched anterior (Ant_10) and posterior (Post_10) electrode trajectories; Table S1: Quantitative comparison between simulation predictions and experimentally recorded field distributions; Table S2: Localization of insular electrode contacts; Table S3: Descriptive statistics, effect sizes, raw *p*-values and Bonferroni-adjusted *p*-values for the primary anterior–posterior comparisons; Table S4: Descriptive statistics, effect sizes, raw *p*-values and Bonferroni-adjusted *p*-values for the matched Ant_10-Post_10 comparisons; Table S5: Quantitative assessment of current-ratio steering across montages.
