# Supplementary material for: Focal and Steerable Temporal Interference Stimulation of the Human Left Posterior Insula: A Proof-of-Concept Validation Using Intracranial Recordings in a Human Cadaver
Source: Bioengineering (Basel). 2026 Sep 10;13(9):1050. doi: 10.3390/bioengineering13091050 (PMC13603869; doi:10.3390/bioengineering13091050)
Supplement: Supplementary file 1 [file bioengineering-13-01050-s001.zip › bioengineering-4493092-supplementary.pdf]

Supplementary Methods: Quantitative comparison of TIP simulation and intracranial recordings

Simulation metrics (TIP.lite)

For classic (two-channel) temporal interference, the modulation envelope magnitude (MEM) at a point x, $T_{max}(x)$, is defined as the maximum, over all orientations , of the smaller projected amplitude of the two channel fields $E_1(x)$ and $E_2(x)$ adapted from [S1].

$$T_{max}(x) = \max_{\|\hat{\mathbf{n}}\|=1} [\min(|\hat{\mathbf{n}}.\mathbf{E_1}(x)|, |\hat{\mathbf{n}}.\mathbf{E_2}(x)|)]$$

TIP.lite computes three exposure-quality metrics from this quantity, evaluated within the target region and the remaining (off-target) brain volume:

Target strength (M1):

$$M1 = median[T_{max}(x)] for\ x \in target\ region$$

Selectivity (M2):

$$M2 = \left({RMS_{target}} \middle/ {RMS_{off-target}} \right)^2$$

where RMS_{target} and $RMS_{off-target}$ are the root-mean-square values of T_{max} within the target region and the remaining (off-target) brain volume, respectively.

Collateral stimulation (M3):

$$M3(\%) = 100 \times (off - target\ volume\ with\ T_{max} > p - th\ percentile\ of\ target\ T_{max}) / (total\ off - target\ volume)$$

with p = 50 (target median), matching the isopercentile setting used in this study.

Experimental analogues

In our assessment for quantitative comparison with the computational predictions, only recording contacts that could be anatomically classified using post-implantation MRI were included (Table S2). Contacts localized within the left insula were assigned to the target set (n = 29), whereas contacts localized in the putamen, claustrum, peri-insular or subcortical regions were assigned to the off-target set (n = 11). The analysis was performed separately for each montage under the 1:1 current condition, corresponding to the condition used for the simulation comparison. For each adjacent-contact bipolar measurement i, the envelope modulation amplitude (A_{env}) was computed as described in Section 2.4. The corresponding field-envelope magnitude $E_{env} = A_{env}/d$, where d=3.5 mm, provides an experimental analogue of T_{max}, measured along the fixed orientation of the bipolar contact pair rather than maximized over all orientations. For this comparison, raw (non-normalized) A_{env} values were used, in contrast to the shaft-normalized values used for the spatial distribution maps in the main text, consistent with M1–M3 above.

Target strength (experimental):

$$M1_{exp} = median[E_{env}] for\ x \in target\ region$$

Selectivity (experimental):

$$M2_{exp} = \left({RMS_{A_{env},target}} \middle/ {RMS_{A_{env},off-target}} \right)^2$$

Collateral stimulation (experimental):

$$M3_{exp}(\%) = 100 \times \left(\#off - target\ differential\ measurments\ with\ E_{env} > \text{median}(E_{env,target})\right) /(total\ number\ of\ off - target\ differential\ measurements)$$

Weighted score

TIP.lite does not publicly disclose the internal algorithm used to combine M1–M3 into a single ranking score from user-set weights. To derive an illustrative experimental analogue, each experimental metric was normalized to its maximum across the three montages and combined using the same relative weights applied during TIP optimization (strength = 0.5, selectivity = 1.0, collateral = 0.5):

$$Weighted\_exp = 0.5\frac{M1}{\max(M1)} + 1.0\frac{M2}{\max(M2)} - 0.5\frac{M3}{\max(M3)}$$

Table S1. Quantitative comparison between simulation predictions and experimentally recorded field distributions.

Data source	Measure	Optimized AP	Optimized lateral	Control AP
**Simulation**	Target strength (V/m)	0.27	**0.29**	0.21
	Selectivity	**4.07**	3.52	2.3
	Collateral stimulation (%)	**4.15**	5.18	9.7
	Weighted TIP	**1.40**	1.24	0.8
**Experimental**	Target strength (V/m)	0.016	0.035	**0.049**
	Selectivity	**1.53**	0.88	0.76
	Collateral stimulation (%)	**12.5**	87.5	87.5
	Weighted_exp	**1.10**	0.43	0.49

Although the magnitude of target strength, selectivity, and collateral stimulation values differed between the simulation and experimental datasets, the relative pattern across montages was preserved for selectivity and collateral stimulation, with the Optimized AP montage outperforming both the Optimized lateral and Control AP montages in the experimental data, consistent with simulation predictions.

The magnitude difference between simulation and experimental values may reflect altered post-mortem tissue properties, freeze-thaw related changes in tissue properties, anatomical mismatch with the computational model, methodological differences between the computational and experimental processing pipelines, and sparse, uneven sampling of target and off-target regions.

Table S2. Localization of insular electrode contacts. Contacts are ordered from deepest to most superficial within each electrode shaft.

Electrode	Contact	tal_x	tal_y	tal_z	Hemisphere	Absolute distance from the midline (mm)	Talairach_label_.
1	Sphere 1	-22	2	6	L	22	Putamen
	Sphere 2	-25	4	6	L	25	Putamen (L)
	Sphere 3	-28	6	6	L	28	Claustrum (L) — .
	Sphere 4	-30	7	6	L	30	Claustrum (L) — .
	Sphere 5	-33	9	6	L	33	Claustrum (L) — .
	Sphere 6	-35	11	6	L	35	Claustrum (L) — .
	Sphere 7	-38	14	6	L	38	Claustrum (L) — .
2	Sphere 8	-29	-11	6	L	29	Insula (BA13, posterior, L) — .
	Sphere 9	-32	-8	6	L	32	Insula (BA13, posterior, L) — .
	Sphere 10	-34	-5	6	L	34	Insula (BA13, posterior, L) — .
	Sphere 11	-37	-2	6	L	37	Insula (BA13, posterior, L) — .
	Sphere 12	-39	1	6	L	39	Claustrum (L) — .
	Sphere 13	-42	5	6	L	42	Insula (BA13, anterior, L) — .
	Sphere 14	-45	8	6	L	45	Insula (BA13, anterior, L) — .
3	Sphere 15	-25	-29	7	L	25	Peri-insular subcortical — dorsal, [illegible]
	Sphere 16	-28	-28	7	L	28	Insula (BA13, posterior, L) — .
	Sphere 17	-31	-26	7	L	31	Insula (BA13, posterior, L) — .
	Sphere 18	-34	-23	7	L	34	Insula (BA13, posterior, L) — .
	Sphere 19	-34	-23	7	L	34	Insula (BA13, posterior, L) — .
	Sphere 20	-37	-21	7	L	37	Insula (BA13, posterior, L) — .
	Sphere 21	-40	-18	7	L	40	Insula (BA13, posterior, L) — .
	Sphere 22	-42	-15	7	L	42	Insula (BA13, posterior, L) — .
	Sphere 23	-45	-14	7	L	45	Insula (BA13, posterior, L) — .
4	Sphere 24	-27	-42	4	L	27	Peri-insular subcortical — dorsal, [illegible]
	Sphere 25	-28	-39	4	L	28	Peri-insular subcortical — dorsal, [illegible]
	Sphere 26	-29	-35	4	L	29	Insula (BA13, posterior, L) — .
	Sphere 27	-32	-30	-3	L	32	Insula (BA13, posterior, L) — .
	Sphere 28	-34	-25	-3	L	34	Insula (BA13, posterior, L) — .
	Sphere 29	-35	-21	-3	L	35	Insula (BA13, posterior, L) — .
	Sphere 30	-38	-16	-5	L	38	Insula (BA13, posterior, L) — .
	Sphere 31	-40	-12	-5	L	40	Insula (BA13, posterior, L) — .
5	Sphere 32	-26	-14	-3	L	26	Insula (BA13, posterior, L) — .
	Sphere 33	-28	-10	-3	L	28	Insula (BA13, posterior, L) — .
	Sphere 34	-32	-7	-3	L	32	Insula (BA13, posterior, L) — .
	Sphere 35	-35	-4	-3	L	35	Insula (BA13, posterior, L) — .
	Sphere 36	-37	0	-3	L	37	Insula (BA13, anterior, L) — .
	Sphere 37	-33	10	-4	L	33	Insula (BA13, anterior, L) — .
6	Sphere 38	-32	7	-8	L	32	Insula (BA13, anterior, L) — .
	Sphere 39	-32	12	-2	L	32	Insula (BA13, anterior, L) — .
	Sphere 40	-27	15	4	L	27	Insula (BA13, anterior, L) — .

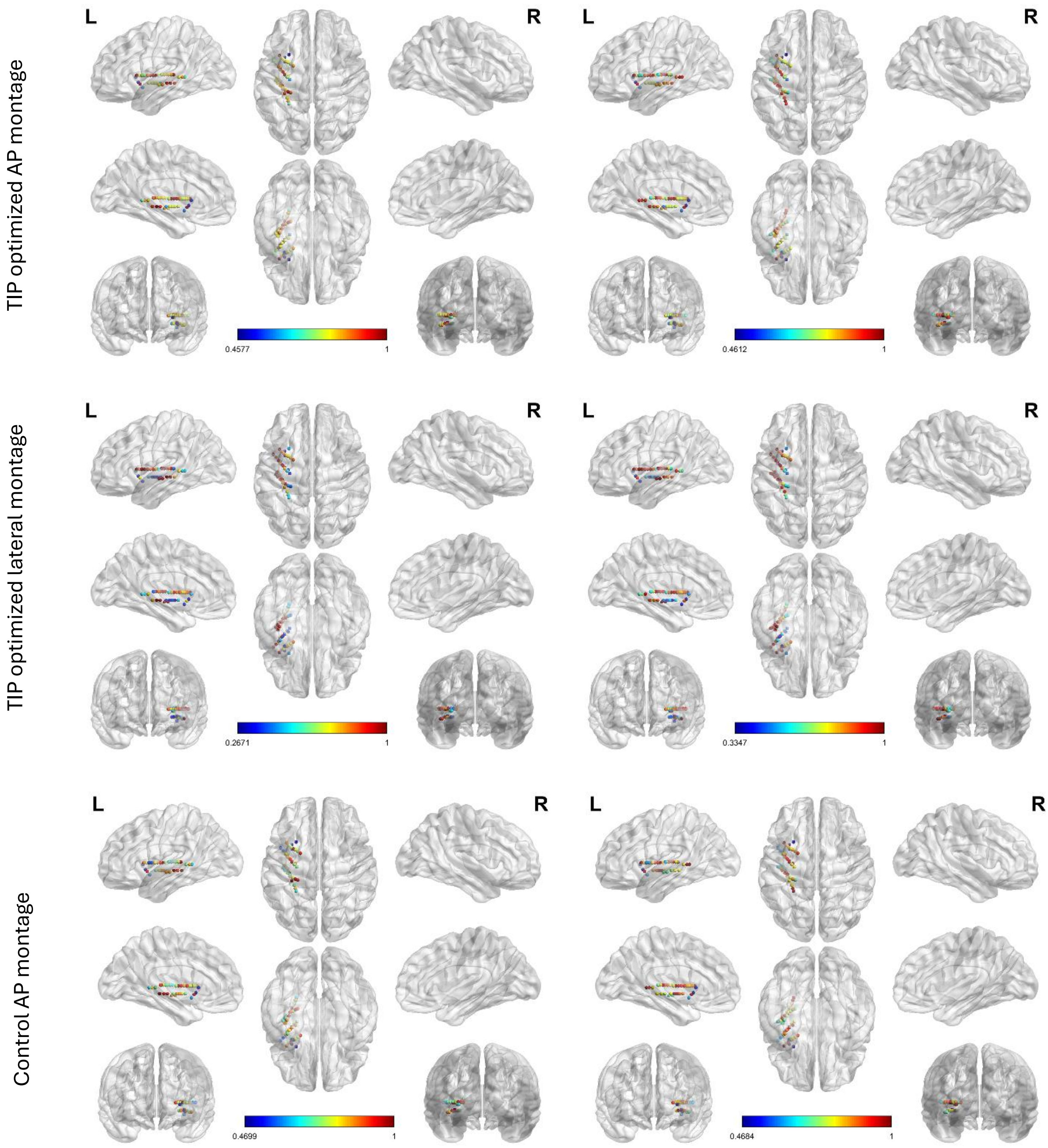

Figure S1. Comparison of normalized absolute-amplitude distributions during TIS and conventional tACS. Glass-brain visualizations generated using BrainNet Viewer in MATLAB [S2] show the TIS absolute amplitude (left column) and conventional 10-Hz tACS absolute amplitude (right column) for the TIP-optimized anterior-posterior montage (top), TIP-optimized lateral montage (middle), and control anterior-posterior montage (bottom). tACS was delivered at 1 mA through each electrode pair. Values were normalized independently within each electrode shaft. Only contacts with available anatomical coordinates are shown (40 of 59 contacts). TIP, temporal interference planning tool; AP, anteroposterior; L, left; R, right.

Table S3. Descriptive statistics, effect sizes, raw p-values, and Bonferroni-adjusted p-values for the primary anterior-posterior comparisons.

Montage	Measure	Depth	n Ant	n Post	Mean Ant	SD Ant	Mean Post	SD Post	Hedges' g	Raw p	Adjusted p
TIP-AP	Envelope	Superficial	9	21	0.7281	0.1984	0.6036	0.1084	-0.8644	0.0340	0.4075
TIP-AP	Envelope	Deep	9	20	0.4690	0.2486	0.7802	0.1908	1.4429	9.7708e-04	0.0117
TIP-AP	Absolute	Superficial	9	21	0.7835	0.2477	0.8332	0.1048	0.3033	0.4405	1.0000
TIP-AP	Absolute	Deep	9	20	0.7205	0.1812	0.8597	0.1159	0.9771	0.0186	0.2232
TIP-Lateral	Envelope	Superficial	9	21	0.7281	0.2519	0.8028	0.1825	0.3547	0.3680	1.0000
TIP-Lateral	Envelope	Deep	9	20	0.5931	0.2078	0.4156	0.2837	-0.6548	0.1048	1.0000
TIP-Lateral	Absolute	Superficial	9	21	0.7170	0.2562	0.8660	0.1560	0.7626	0.0591	0.7097
TIP-Lateral	Absolute	Deep	9	20	0.6723	0.2226	0.6363	0.2388	-0.1496	0.7045	1.0000
Control-AP	Envelope	Superficial	9	21	0.6195	0.1235	0.8023	0.1258	1.4206	0.0010	0.0123
Control-AP	Envelope	Deep	9	20	0.7337	0.2037	0.8298	0.1513	0.5538	0.1672	1.0000
Control-AP	Absolute	Superficial	9	21	0.7044	0.1902	0.7412	0.1284	0.2408	0.5396	1.0000
Control-AP	Absolute	Deep	9	20	0.7540	0.2019	0.8460	0.1048	0.6357	0.1148	1.0000

Note. Ant, anterior; Post, posterior; SD, standard deviation. n represents the number of contact-level measurements within the anterior and posterior trajectory groups. Raw p-values are from two-sided two-sample t-tests. Adjusted p-values were Bonferroni-corrected across the 12 comparisons. Positive Hedges' g values indicate Post > Ant.

Table S4. Descriptive statistics, effect sizes, raw p-values, and Bonferroni-adjusted p-values for the matched Ant_10-Post_10 comparisons.

Montage	Measure	Depth	n Ant	n Post	Mean Ant	SD Ant	Mean Post	SD Post	Hedges' g	Raw p	Adjusted p
TIP-AP	Envelope	Superficial	5	5	0.7892	0.2397	0.6070	0.0717	-0.9302	0.1421	1.0000
TIP-AP	Envelope	Deep	5	5	0.3298	0.0744	0.7547	0.2104	2.4317	0.0028	0.0333
TIP-AP	Absolute	Superficial	5	5	0.9465	0.0780	0.7900	0.1206	-1.3915	0.0408	0.4899
TIP-AP	Absolute	Deep	5	5	0.7946	0.0438	0.8492	0.1176	0.5563	0.3586	1.0000
TIP-Lateral	Envelope	Superficial	5	5	0.8608	0.1027	0.9116	0.0608	0.5441	0.3688	1.0000
TIP-Lateral	Envelope	Deep	5	5	0.6758	0.0579	0.4902	0.2371	-0.9715	0.1274	1.0000
TIP-Lateral	Absolute	Superficial	5	5	0.8479	0.1116	0.9520	0.0416	1.1168	0.0863	1.0000
TIP-Lateral	Absolute	Deep	5	5	0.7880	0.0467	0.7086	0.2031	-0.4867	0.4190	1.0000
Control-AP	Envelope	Superficial	5	5	0.6397	0.0368	0.8801	0.1335	2.2178	0.0047	0.0559
Control-AP	Envelope	Deep	5	5	0.8101	0.1471	0.7660	0.2080	-0.2213	0.7085	1.0000
Control-AP	Absolute	Superficial	5	5	0.8027	0.0556	0.8738	0.0591	1.1193	0.0857	1.0000
Control-AP	Absolute	Deep	5	5	0.8450	0.1100	0.8273	0.1437	-0.1255	0.8316	1.0000

Note. Ant, anterior; Post, posterior; SD, standard deviation. n represents the number of contact-level measurements within the Ant_10 and Post_10 trajectories. Raw p-values are from two-sided two-sample t-tests. Adjusted p-values were Bonferroni-corrected across the 12 comparisons. Positive Hedges' g values indicate Post > Ant.

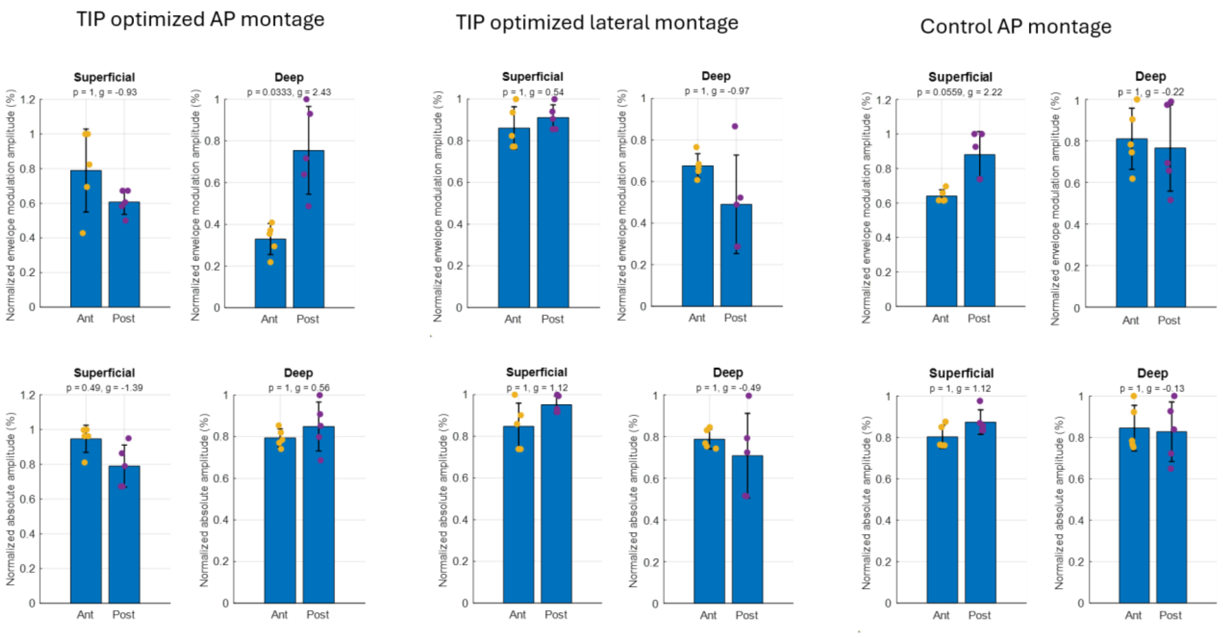

Figure S2. Comparison of normalized envelope modulation amplitude (top row) and normalized absolute amplitude (bottom row) between anterior (Ant) and posterior (Post) electrode trajectories restricting to matched anterior/posterior pairs (Ant_10 vs Post_10) only. For each montage, analyses were performed separately for superficial contacts (left panel) and deep contacts (right panel), defined as the superficial and deepest 50% of contacts along each electrode trajectory, respectively. Bars represent mean ± SD, and individual points represent contacts. P-values were Bonferroni-adjusted across the 12 comparisons. Effect sizes are reported as Hedges' g, with positive values indicating Post > Ant. A total of 10 anterior and 10 posterior contacts were included. Ant, anterior; Post, posterior; TIP, temporal interference planning tool; AP, anteroposterior; SD, standard deviation.

Table S5. Quantitative assessment of current-ratio steering across montages.

Panel A. Steering effect magnitude at constant total current

Montage	Ant/Post TI 1:2 (%)	Ant/Post TI 2:1 (%)	Constant 2.4 mA change (pp)	Constant 2.4 mA relative change (%)	Ant/Post TI 2:3 (%)	Ant/Post TI 3:2 (%)	Constant 2 mA change (pp)	Constant 2 mA relative change (%)
Optimized AP	90.66	75.43	-15.22	-16.79	82.93	77.29	-5.63	-6.79
Optimized lateral	107.84	107.16	-0.68	-0.63	108.12	115.79	7.67	7.09
Non-optimized AP	97.92	106.41	8.49	8.67	106.99	95.37	-11.62	-10.86

Panel B. Descriptive regression and rank-correlation statistics

Montage	Slope (pp/unit ratio)	Slope 95% CI lower	Slope 95% CI upper	R^2	Spearman rho	Spearman p	Number of conditions
Optimized AP	-8.77	-15.99	-1.56	0.83	-1.0000	0.0167	5
Optimized lateral	1.28	-8.92	11.49	0.05	0.0000	1.0000	5
Non-optimized AP	2.30	-20.98	25.58	0.03	-0.1000	0.9500	5

Note. Ant/Post, anterior-to-posterior modulation ratio; AP, anterior-posterior; pp, percentage points; CI, confidence interval. Constant 2.4 mA change = TI 2:1 minus TI 1:2; both conditions used a total current of 2.4 mA. Relative change = 100 multiplied by the endpoint change divided by TI 1:2. Constant 2 mA change = TI 3:2 minus TI 2:3; both conditions used a total current of 2.0 mA. Slope units are percentage points per unit increase in I1/I2. Regression statistics are descriptive because only five current-ratio conditions were evaluated.

S1. Grossman, N.; Bono, D.; Dedic, N.; Kodandaramaiah, S.B.; Rudenko, A.; Suk, H.-J.; Cassara, A.M.; Neufeld, E.; Kuster, N.; Tsai, L.-H.; et al. Noninvasive Deep Brain Stimulation via Temporally Interfering Electric Fields. *Cell* **2017**, *169*, 1029-1041.e16, doi:10.1016/j.cell.2017.05.024.

S2. Xia, M.; Wang, J.; He, Y. BrainNet Viewer: A Network Visualization Tool for Human Brain Connectomics. *PLoS One* **2013**, *8*, e68910, doi:10.1371/journal.pone.0068910.
